# Supplementary material for: Are carbon emissions trading and green financial instruments synergistic? -Comprehensive quantitative research based on content analysis
Source: PLoS One. 2024 Mar 7;19(3):e0298601. doi: 10.1371/journal.pone.0298601 (PMC10919601; doi:10.1371/journal.pone.0298601)
Supplement: S1 Data — (ZIP) [file pone.0298601.s001.zip › Data/地级市Z_F工作报告.zip!/║■─╧/╧μ╠╢╩╨/└┤╫╘╣1⁄2╓┌║┼í╛┬φ┐╦╩2╛▌═°í┐_2017.docx]

各位代表：

现在，我代表市人民政府向大会报告工作，请予审查，并请各位政协委员和其他列席会议人员提出意见。

一、２０１６年和本届政府工作回顾

２０１６年，是“十三五”的开局之年。面对经济下行压力，在省委省政府和市委的坚强领导下，我们紧紧依靠和团结全市人民，坚持稳住、调优、进好，统筹全局弹好钢琴，攻堡垒、解难题、创新局、图长远，各项主要目标任务如期完成，全面小康社会建设胜利达标。

（一）突出产业发展，经济增长实现了稳中有进。牢牢抓住建设国家自主创新示范区重大机遇，围绕打造“智造谷”、构建“１＋４”特色产业体系，统筹实施“三去一降一补”、产业补链强链、重点企业调度、中小企业帮扶、县域经济发展、园区二次创业等系列措施，稳住了工业主阵地，促进了产业转型升级。规模以上工业企业达到９３０家。“１＋４”产业实现规模工业增加值５９６亿元，占全市规模工业增加值比重达７０％。装备制造产业实现产值８９０亿元，汽车产业实现产值２８０亿元。高新技术产业产值突破２０００亿元。吉利、泰富产值分别达１４０亿元、１８０亿元。湘潭县稳增长工作受到国务院通报表扬。步步高集团迈入中国５００强企业，综保区跨境电子商务直购平台启用，举办第五届湖南省电子商务大会，规模以上服务业营业收入达８０亿元。成功实施韶山核心景区交通换乘。承办全国旅行社协会换届大会，中国５００强旅行社齐聚湘潭。全年旅游综合收入达２７５亿元。国家现代农业示范区建设加快推进，全市农林牧渔业增加值达１５０亿元，规模以上农产品加工企业产值与农业总产值的比值达２．６∶１。非公经济对投资贡献率达７０％，税收贡献率达６７％，就业贡献率达８８％。全年实现地区生产总值１８４０亿元（预计数，下同），同比增长８％；规模工业增加值８６０亿元，增长６．８％；固定资产投资１９４７亿元，增长１５％；完成财政总收入１８６．４３亿元，增长６．２８％；社会消费品零售总额５８３亿元，增长１２％；三次产业结构调整为８∶５３．１∶３８．９，保持了稳中有进、稳中向好的发展势头。

（二）建设精美湘潭，城乡建设掀开了崭新篇章。以创建全国文明城市为引领，启动精美湘潭行动计划，全面推进城市基础设施建设、美丽乡村建设和市容环境“三化两拆两改”。板霞路、湘江风光带铁路桥至大埠桥段全面完工，莲城大道九华段、熙春北路延长线、芙蓉大道等道路主路面通车，河东大道路面完成改造，城市主干道承载功能明显增强。加速昭华大桥、杨梅洲大桥建设，“一江九桥”格局初步成形。碧泉湖、仰天湖、百亩湖建设顺利推进，爱劳渠、争光渠、丰收渠等黑臭水体有效整治，城市水系治理成效初显。火车站南广场、沪昆北站广场、长株潭城际铁路站场按期投入使用，湘潭以全新的姿态迈入普铁、高铁、城铁“三铁时代”。累计投入７亿元建设美丽乡村，梅林桥、银田、昭山等美丽乡村示范片初见成效，农村环境卫生综合整治持续深化，长效机制逐步建立。完成窑湾文化旅游一期景观工程建设。一大桥桥头、砂子岭转盘等１７处城市堵点基本打通。１９家机关院落完成拆墙透绿，实现还绿于民。扎实开展市容环境“五项整治”，市城区拆除违法建设７５万平方米，市容市貌明显改观。完成大同花苑、金霞苑等４８个老旧小区提质改造，富都花园等５３个物管盲区实现物业管理覆盖，消除照明暗区７９０处，打造了风车坪等精美示范社区，“水通、路平、灯亮”的社区新面貌逐步呈现。湘潭人民对建设美好家园的参与度、认同感全面增强，追求美好生活的脚步更为有力，铿锵前行的步伐更加自信。

（三）狠抓有效投资，项目建设取得了重大进展。瞄准“大项目、大企业、大产业”，集中精力抓审批、抓开工、抓储备。全力推进重大项目落地见效，泰富重装二期、屹丰汽车零部件二期、崇德科技生产基地等建成投产，吉利ＳＵＶ生产平台、桑顿新能源２．５期进展顺利，万达广场、金阳商贸城一期、住总家家美等商业综合体建成开业，盘龙大观园二期顺利推进，山市晴岚项目正式开工，全年组织实施重点产业项目１７４个，完成投资４４０亿元，投资对经济增长的贡献率达到５５％。举办了京洽会、沪洽周专题招商、对接东盟商机推介会等重大活动，入选２０１６中国十佳最具投资营商价值城市，舍弗勒湘潭基地、长株潭Ｆ２国际赛车文化产业园、“最忆韶山冲”文旅综合体、湖南健康产业园等一批重大项目成功落户，引进世界５００强企业１３家，国内５００强企业１４家，全年累计签约亿元以上项目９２个，总投资２３６２．４亿元。用地保障实现历史性突破，市城区征收国有土地上房屋签约１１２万平方米，是上年的５倍；征收集体土地上房屋签约１１１万平方米，是上年的３倍；腾交土地７３２４亩，是上年的９倍，保证了重点项目用地所需，坚定了企业投资信心。

（四）坚持深化改革，发展活力得到了有效释放。全面推进供给侧结构性改革，实施直供电改革、报建费减免、社保费下调等举措，“三去一降一补”重点任务成效显著。聚焦融资难、融资贵问题，成立了总规模达２３２．０３亿元的城市发展、产业发展等８支基金，取得了交通银行等７家银行共计１３００亿元的授信支持，新增社会融资规模同比增长６０．４２％，全市各项贷款加权平均利率同比下降１６．９１％，政府性债务置换节约利息成本２．２亿元。湖南股权交易所湘潭分所挂牌成立，金融生态评估位列全省第二。引入社会资本参与棚户区改造和重大基础设施建设，新型智慧城市建设、万楼片区城中村改造等８个项目被列入第三批国省ＰＰＰ示范项目。房地产市场去库存效果明显，市本级新建商品房剩余可售量３３１．８万平方米，较上年底减少１２８．７万平方米。全面推进农村土地承包经营权确权登记颁证工作，全市农村耕地流转率达４７．５％，农民合作社达１５７５家、家庭农场达６１０户。积极推进国企改制改革，组建交发集团、潭房集团、湘盾集团，完成湘钢改水、改电工作，为全省国企办社会职能分离移交提供了可复制、可推广的工作经验。清理规范中介服务事项７１项，行业协会商会脱钩２４８家，下放园区管理权限１１９项，在全省率先开展委托乡镇执法和承担行政职能事业单位改革试点。

（五）落实以民为本，发展成果体现了惠民安民。民生保障水平稳居全省前列。公共财政民生支出比重保持在６４％以上。完成３０８７５名建档立卡贫困人口预脱贫和６５个省定贫困村摘帽。城乡居民人均可支配收入分别达３．１６万元和１．６７万元，同比增长８．０％、８．５％。全市新增城镇就业５．２４万人，新增农村劳动力转移就业１．４万人。全面完成义务教育合格学校建设，各县市区均通过“全国义务教育发展基本均衡县”评估验收。推进学区联盟集团化办学，高中阶段毛入学率和普高学考质量全省领先。实施乡村教师支持计划、农村中职教育攻坚计划，推进国家级职业教育现代学徒制试点，建成全省首家示范性综合实践基地。置换非义务教育阶段学校债务２．４７亿元。城乡居民最低生活保障标准大幅提高。在全省率先实现农村低保标准与扶贫标准“两线融合”。整合城乡居民基本医疗保险，全面启动城市公立医院综合改革。成功创建全国基层中医药工作先进市，获批第一批国家级医养结合试点城市、社区和居家养老服务试点城市。文化惠民政策全面落实，群众文体活动蓬勃开展。成功举办市第十二届运动会，率先创建全省首个全民健身示范城市，实现奥运奖牌零的突破。全国道德模范与身边好人现场交流活动在湘潭举行。“两房两棚”工作成绩显著，改造农村危房５５６０户，１．４万户棚户区居民喜迁新居，棚户区改造经验受到住建部肯定并予以推介。投入公共自行车５５００辆，使用人次超过４９３万，市民绿色出行更加便利。竹埠港２８家化工企业的搬迁、拆除工作全面完成，水府庙库区２．８万口网箱全部取缔，湘江流域保护和治理“一号重点工程”成效明显，湘江水质稳定达标，空气质量达标率同比上升４个百分点。抓好粮食收购和储备，实施“放心粮油”工程。道路交通管理进一步加强，治安防控网全面覆盖，社会治安不断好转，公众满意度明显上升，综治民调、安全生产、治超治限、质量工作等考核排名全省第一。

（六）加强自身建设，政府工作呈现了新的面貌。有效发挥政府党组领导核心作用，以“全面从严治党”统领政府各项工作，扎实开展“两学一做”学习教育。加大正面激励力度，引导公职人员更富担当精神和改革魄力，以“舍我其谁”的责任感、“只争朝夕”的紧迫感、“建功有我”的使命感，将湘潭的发展重担扛牢在肩。扎实推进精美湘潭、园区发展、创新创业等八大行动计划，形成了干事创业、攻坚克难的工作局面。全面推广“一线工作法”，各级领导和广大干部，人在现场，心在现场，干在现场，评在现场，执行力明显增强。全面推行精准考核，坚持结果考核和过程考核并重，考核方案与实施方案同步制定、同步执行、同步反馈，强化了时间节点管理。严格执行重大事项集体研究制度、会议审批制度和“三重一大”决策制度，决策、审批、执行更有效率，会务、办文、议事更讲规则。坚持市长办公周例会和重点任务市长交办函制度，实施市级领导领办重点产业项目、对口帮扶企业、联系棚户区改造等中心工作。集中研究化解了一批企业和群众反映强烈的历史遗留问题。

民族宗教、妇女儿童、青少年、老龄、工会、科协、社科联、侨联、文联、残联、林业、农机、韶灌、移民、气象、地震、水文、广播电视、国安、对台、档案、市志、公积金管理、工商联、供销合作等工作取得新进步。军民融合发展持续深化，国防动员、国防教育和国防后备力量建设不断加强，荣获全国双拥模范城市“三连冠”和全国人民防空先进城市。

各位代表，本届政府工作的四年，是经济发展进入新常态、经济结构深入调整的四年。四年来，我们始终保持战略定力，抓住关键环节，坚持稳中求进，既打好歼灭战，又坚持持久战，着力培育内生增长动力，全市地区生产总值年均增长９．８％，财政总收入年均增长１１％；固定资产投资年均增长２３．４％，社会消费品零售总额年均增长１２．９％。四年来，全市形成了稳增速、优结构、转动能的良好发展势头。

——稳步推进发展升级。湘潭纳入国家自主创新示范区等２０多项国家级平台，“智造谷”纳入“中国制造２０２５”国家战略，四个特色产业园纳入全省战略布局。“一钢独大”局面彻底改变，“１＋４”产业蓬勃发展。湘潭经开区、高新区成为千亿园区。综保区封关运行，韶山出入境检验检疫局入驻。湘潭县、湘乡市成为全省经济强县，湘潭县、韶山市成为全省第一批特色县域经济重点县，雨湖区成功获批国家新型工业化军民结合产业示范基地，雨湖工业集中区晋升省级经济开发区，岳塘经开区现代服务业集聚势头强劲。湘电、湘钢、电化等传统企业加快技改，竹埠港、锰矿、湘乡城西等老工矿区全线转型。“海牛”深海钻机等技术跻身世界一流。国家工矿电传动车辆质检中心建成启用。岳塘国际商贸城、一力物流公路港、中国网库等项目落地开工，商贸物流、会展经济、电子商务蓬勃发展。都市农业等新业态逐步显现，农业产业化来势喜人。平台引领、园区支撑、多点发力的发展格局逐渐形成。

——统筹推进城乡建设。完成了城市总规、土地利用总规修编，编制了中心城区控规及配套专项规划，城乡架构基本框定，“一核五城”完成布局。河东城区功能大幅完善，河西旧城改造有序推进，九华、昭岳、天易等新城片区初具规模，全市城镇化率由５４．０２％提高到５９．５％。建成一批重点公共设施和宜居项目，湖湘公园完成扩容提质，九华湖、木鱼湖公园等建成开放。沪昆高铁、长株潭城铁开通运行，潭州大道、昭云大道等６条城际主干道建成通车，“七纵七横三支”综合路网基本成型。成功回购四大桥经营权并取消收费，市民过江更加便捷。调整优化公交线路５５条，开通全省首条城乡客运一体化示范公交线路。建成农村公路３０２９公里，通信、供水等城乡基础设施加速融合。银田、棋梓、花石等特色小城镇建设初显成效，壶天镇入选全国特色景观旅游名镇，壶天村、顶峰村入选中国传统村落名录。圆满完成首轮农村环境卫生综合整治。全面完成“两型社会”综合配套改革试验区第二阶段改革任务。

——全面推进民生事业。城乡人均可支配收入年均分别增长８．９％和１１．９％。通过产业扶贫、劳务对接、金融扶贫、易地搬迁等途径，实现９万建档立卡农村贫困人口精准脱贫。累计新增城镇就业２３．４３万人，农村劳动力转移７．２８万人，获评全国创业先进城市。廉租房和公租房并轨运行，累计解决１２．１万户困难家庭住房问题，改造农村危房１．６７万户。市城区被征地农民社保遗留问题得到解决。社会保障体系持续扩面提标，大病保险全面覆盖，新增养老床位６５００张，基本养老服务补贴覆盖率达到１００％。农村最低生活保障标准提高了１．６倍。教育事业均衡发展，建成农村公办幼儿园６２所，建成义务教育合格学校５１２所，新增义务教育学位１２０６９个，全面取消中小学择校。成功举办第四届中国（湘潭）齐白石国际文化艺术节，大型花鼓戏《齐白石》荣获第五届湖南艺术节田汉大奖。《中国出了个毛泽东》实景演出广受好评。党史馆、博物馆和规划展示馆顺利开馆。计生工作连续八年保持全省模范。安全生产形势保持总体稳定，湘潭县、韶山市成为全省安全生产示范县市。法治湘潭、平安湘潭建设持续推进，综治民调连续四年名列全省前茅。

——切实推进简政放权。深化行政审批制度改革，市本级取消行政审批项目２９项，承接省级经济社会管理权限４７项，政务大厅共进驻行政审批单位４４家、审批及服务事项３７１项，行政审批事项全部实现在线办理，完善了投资项目大并联审批、全流程服务机制，“放管服”工作经验在全国推介。市本级政府投资项目全面推行预算、结算、决算“三审制”。“营改增”全面铺开。商事制度改革稳步推进。全面完成市县政府机构改革和事业单位分类改革。顺利完成乡镇合并、建制村合并。完成了党政机关公务用车制度改革。公共资源交易中心、不动产登记中心有效运行。市应急联动指挥中心投入使用，实现１１０报警服务平台和“１２３４５”市长热线合署运行。人大代表议案、建议，政协委员提案办理满意率每年均达到９８％以上。

各位代表，过去四年成绩的取得，是市委正确领导，市人大依法监督，市政协全力支持，全市人民共同奋斗的结果。在此，我代表市人民政府向全市人民，向各民主党派、工商联、人民团体和各界人士，向驻潭部队、武警官兵和公安民警，向所有关心支持湘潭发展的朋友，表示衷心的感谢和崇高的敬意！

总结成绩的同时，我们必须清醒认识到面临的挑战和存在的问题：旧的增长动力深度调整，新的发展动能尚未完全释放，推进供给侧结构性改革任务艰巨，稳增长、调结构压力巨大；经济发展质量不高，实体经济比较困难，民间投资偏少，抓收入、保支出困难不小；园区规模工业总量不大，县域经济发展不足，镇村经济十分欠缺，培优势、补短板尤为迫切；精美湘潭建设、民生保障水平、干部作风能力、政府自身建设等与人民群众的期盼仍有较大差距。这些问题，我们必须高度重视，采取有效措施加以解决。

二、未来五年的主要目标和任务

未来五年，是我市建设“伟人故里、大美湘潭”，率先迈向基本现代化的关键五年。在“一带一路”、长江经济带、长江中游城市群等国家重大发展战略中，湘潭的发展空间极大拓展、政策资源极为丰富；在省委省政府大力实施“创新引领、开放崛起”战略，推进“三个着力”“四大体系”“五大基地”总体部署，打造长株潭核心增长极的发展格局中，湘潭地处战略要冲、发展核心；在湖南重塑经济空间，厚植发展优势，奋力走在中部崛起前列的伟大进程中，湘潭责任重大，机遇空前。我们必须砥砺奋进，率先发展，为建设富饶美丽幸福新湖南作出应有的贡献。

各位代表，机遇总是垂青勇于竞争的人，我们要对标一流，精进前行，蹄疾步稳踏上新一轮“跨越发展、稳步赶超”的豪迈征程。未来五年，我们将深入贯彻党的十八大及十八届三中、四中、五中、六中全会精神和习近平总书记系列重要讲话精神，全面落实省第十一次党代会战略部署，坚持稳中求进工作总基调，牢固树立和贯彻落实新发展理念，适应把握引领经济发展新常态，坚持以提高发展质量和效益为中心，坚持以深化供给侧结构性改革为主线，突出抓好产业项目、园区发展、精美湘潭、文化旅游四个板块，着力推进民生改善、深化改革、社会治理、自身建设四大任务，一个时期一个重点，一个阶段一个专题，把市第十二次党代会确定的发展目标、把人民群众对美好生活的热切期盼分解到年度，细化到项目，落实到成效。到２０２１年，我们要实现以下基本目标：

——率先迈向基本现代化的经济实力。以智能制造业为主导的产业新格局初具规模，规模工业增加值达到１４００亿元以上，占地区生产总值比重保持在４９％左右；服务业增加值达到１２００亿元以上，占地区生产总值比重保持在４２％左右；金融业增加值占地区生产总值比重达４．３％以上。地区生产总值达到３０００亿元左右，财政总收入达到３００亿元左右，人均地区生产总值达１０万元以上，综合经济实力稳居全省第一方阵前列。

——率先迈向基本现代化的创新能力。科技文化创新体系活力迸发，人才和科技进步对经济增长贡献率显著增强。高新技术产业增加值达到１０００亿元以上，占地区生产总值比重３６％以上；研发经费支出达到８５亿元以上，占地区生产总值比重３％以上；万人发明专利拥有量８件以上；五年累计新增科技成果３００项以上，实现技术合同交易额１００亿元以上。

——率先迈向基本现代化的开放水平。初步形成具有强大吸引力的开放型经济体制与全方位开放格局，年度外贸进出口额达到５２亿美元以上，外贸依存度达到１２％以上；年度引进市外资金７００亿元以上，引进世界５００强企业３５家以上，设立境外企业２５家以上，对外经济合作营业额占地区生产总值比重达到３％以上；年接待国内外游客１２００万人以上。

——率先迈向基本现代化的城乡品质。形成独具魅力的新型城镇体系，城镇化率达６６％以上，城市空气质量优良率达８０％以上，建成区绿地率达３６．２９％以上，全市森林覆盖率保持在４６．３％以上，水功能区水质达标率、生活垃圾无害化处理率和城镇污水达标处理率均达９５％以上，争创全国文明城市、国家森林城市、国家卫生城市、国家交通管理模范城市、生态文明示范市、国家历史文化名城。

——率先迈向基本现代化的人的全面发展。就业和收入水平显著提高，城乡居民人均可支配收入分别达４．８万元、２．６万元以上。新增劳动力人均受教育年限达１４年，高等教育毛入学率达到５０％以上，在校大学生达１７．８万人。文化湘潭、智慧湘潭、健康湘潭、法治湘潭、平安湘潭等建设富有成效。全面建成法治政府，群众对法治建设满意度达８０％以上。

以上述目标为统领，我们要精准发力，重点主攻：

（一）创新引领，培育现代产业体系。深入推进长株潭国家自主创新示范区创建工作，建设智能制造科技创新中心，组建产业技术创新联盟，加快市产业创新研究院及其所属专业院所建设。新增２家以上国家级企业技术中心和工程技术研究中心，建立２０个以上创新创业苗圃与企业孵化器。整合高校、企业、科研院所创新资源，加快形成产业创新链条。围绕创建“中国制造２０２５”试点示范城市群，加快湘潭“智造谷”建设，实施企业智能化改造升级滚动计划，力争到２０２１年建成２０家以上智能工厂，全市大中型企业关键工序和核心装备数控化率达６５％以上，推进湘潭制造向中高端迈进。发展“１＋４”特色产业，实施企业成长“１５８”工程，力争到２０２１年，成功培育“百亿”企业１０家以上、“十亿”企业５０家以上、亿元企业８００家以上，实现汽车产业过千亿元、装备产业过两千亿元。加快把韶山建成世界知名旅游目的地和“锦绣潇湘”核心品牌，把“千里湘江第一湾”建成全国知名经典景区，打造山市晴岚、盘龙大观园、长株潭Ｆ２赛道等旅游新地标。加强红色文化、名人文化、湖湘文化资源的深度保护和开发，发展文化创意产业，建设韶山文化演艺创意聚集区、窑湾白石书画艺术创意聚集区、碧泉湖工业设计创意聚集区。推进国家现代农业示范区建设，大力发展都市农业、观光农业、精致农业，培育粮油、畜禽、槟榔三个２００亿和蔬菜水果、花卉苗木、湘莲三个１００亿全产业链集群。

（二）开放崛起，构筑开放发展高地。深入对接国家“一带一路”、长江经济带等重大战略，着力引进汽车制造、装备制造、文化旅游等链群配套型企业；积极推进湘钢、吉利、泰富等重点企业产能出海，组织企业参加汉诺威工业展等国际展会。推进公铁水联运，建设九华铁路综合物流园，完成湘黔铁路东西货站搬迁。加快综保区申报进口肉类指定口岸资质，进一步完善口岸体系，营造通关便利环境。发挥园区开放发展主阵地作用，实现湘潭高新区、经开区工业总产值均超过２０００亿元，昭山示范区创建为国家级文化产业示范园区，支持天易示范区晋级国家级园区，争取综保区纳入申报中国（湖南）自由贸易试验区范围。加快打造长株潭供应链核心枢纽，推动岳塘区建设长株潭核心商务区；推动湘潭经开区和雨湖区进入湘江新区带动范围。重点发展建设路口等五大核心商圈。支持步步高集团实现销售收入过千亿。支持韶山市、昭山示范区创建国家全域旅游示范区，以韶山为核心创建国家红色旅游融合发展示范区、红色旅游国际合作示范区。推动湘潭县、湘乡市迈进全国百强县。

（三）品质至上，建设精致精美家园。深入推进国家新型城镇化综合试点工作。坚持集约精当、精明增长原则，落实中心城区“一核五城”和湘乡、韶山两个次中心建设布局，建好一批中心镇、重点镇、特色镇。高标准建设九华、昭岳、万楼、天易等重点片区，加快与长沙、株洲融城发展。实现２０％以上的行政村建成美丽乡村示范村，２０％以上的乡镇、街道全域达到美丽乡村建设要求，韶山市建成全域美丽乡村示范市。立足城际对接、城乡一体，建成“九纵十三横”干线公路网和“三纵三横三支”高速路网，建成城市一环，贯通城市二环，围合城市三环。完成昭华大桥、杨梅洲大桥、下摄司大桥建设。建成北连鹤岭，南接花石，总长达１００公里的建设路南北大走廊。努力推进“长湘株”轨道交通建设。全面完成城区黑臭水体治理。建成７７．４公里干支线地下综合管廊。规划建设河东绿色生态景观轴，实现人均享有公共绿地１１平方米以上，创建国家生态园林城市。让精美的城市、美丽的乡村，共同丰富“大美湘潭”的精彩底色。

（四）民生优先，全民共享发展成果。坚持以人民的获得感来衡量发展成果，民生支出占财政支出比重逐年提高。实施居民收入倍增计划，确保居民人均可支配收入增速跑赢地区生产总值增速。推动大众创业、万众创新，促进更高质量就业，城镇登记失业率控制在４．２％以下。实施全民参保计划，完善社会保障体系。持续推进保障性安居工程。扎实推进精准扶贫和精准脱贫，确保如期脱贫、脱贫不返贫。投入８亿元以上，实施惠及全市农民的电网改造三年行动计划，彻底解决农村低电压引起的电器使用问题，满足农村特别是边远地区人民群众日益增长的用电需求。加大文化事业投入，推进文化惠民工程，建成“一馆五中心”和公共文化服务网络。坚持教育优先和均衡发展，推进教育领域综合改革，教育发展水平保持全省先进行列，基本建成教育强市。深化医药卫生体制改革，推行分级诊疗，加快基层医疗卫生服务体系建设。广泛开展全民健身运动，普遍提升群众健康水平。提高人口素质，优化人口结构，促进人口长期均衡发展。让人民群众享有更优质的学位，更便捷的就医，更舒适的环境，更全面的保障，让父老乡亲过上接近中等发达地区水平的美好生活。

各位代表，建设“伟人故里、大美湘潭”，率先迈向基本现代化，是市第十二次党代会对全市人民的庄严承诺，是政府必须坚决落实的重大责任。目标已经确定，蓝图已经绘就，我们必须矢志不移，毫不松懈，不翻新口号，不走偏走样，不好大喜功，不急功近利，驰而不息，久久为功，直到目标的圆满实现。

三、２０１７年工作重点

２０１７年是新一届政府的开局之年。根据中央、省委、市委经济工作会议精神和未来五年目标任务，全年的主要预期目标为：地区生产总值增长８．５％左右，固定资产投资增长１５％左右，规模工业增加值增长７％以上，财政总收入增长６％以上，社会消费品零售总额增长１２％左右，进出口总额增长１２％左右，城乡居民人均可支配收入分别增长８．０％和８．５％以上。地区生产总值过２０００亿元、固定资产投资过２０００亿元，财政总收入过２００亿元。重点抓好七个方面工作：

（一）突出产业振兴，培优经济增长动力。推进新型工业化。实施工业兴市、工业强市战略，坚持优化存量、引导增量、主动减量，全面启动“中国制造２０２５”试点示范工作。选择一批优质企业，进行生产环节的数字化、网络化改造，提升流程管理能力，构建智能制造体系。开展“产业补链强群年”活动，坚持“一链一策”，启动汽车千亿产业行动计划和装备制造千亿产业倍增计划。申报国家军民融合示范区，推动军民融合产业发展。实施“１１５５”创新引领科技行动，鼓励校企共建，促进院校科研成果就地转化。加快力合长株潭科技领航创新基地、院士创新产业园等项目建设，引进、培育领军型人才、高技能人才和职业经理人，推进实施人才强市战略。大力弘扬“工匠精神”，实施“精进质量”计划，创建全国质量强市示范城市。强化园区主阵地。开展“百强企业进湘潭”“大型项目入园区”等活动，国家级园区引进年投资３０亿元以上产业项目１个，或２０亿元以上项目２个；省级园区引进年投资２０亿元以上产业项目１个，或１０亿元以上项目２个。支持湘潭经开区推进增量配电改革和园区系统性改革试点。支持湘潭高新区改革管理体制，开展“一区多园”试点。继续推进审批权力下放，实行“缴费清单”制度。发展现代服务业。加快岳塘国际商贸城、金阳商贸城等重大商贸项目建设，完善建设路口、砂子岭核心商圈和福星金融商务区功能配套。复制自贸区改革试点经验，加快将综保区建成多功能贸易融合发展示范基地。着力提升房地产开发品质，通过举办房源展示会、出台优惠政策等手段，激活本地需求，吸纳周边需求，形成房产消费洼地效应。完善农产品流通骨干网络，开展肉菜流通追溯体系国家试点。强化粮食仓储物流体系建设，深入推进“放心粮油”工程。

（二）围绕宜居宜游，提速精美湘潭建设。推进城市基础设施建设。完成芙蓉大道、莲城大道等城市出入主通道整治，完成韶山路、建设路等城市主干道提质，加快昭华大桥、杨梅洲大桥建设，启动下摄司大桥建设。完成五座大桥交通优化及桥头公园建设。基本建成湘江风光带、碧泉湖公园、仰天湖公园等城市景观。加快“一馆五中心”建设，建好市档案馆。推进护潭西路、新马路等地下综合管廊和鹤岭、银田污水处理厂建设，整治江麓西干渠、爱劳渠、护潭河等黑臭水体。加快河西、河东滨江片区等９大重点棚户片区改造，启动宝塔片区、万楼片区等城中村项目。建设河东、河西公交客运总站。深化城市精细管理。加大“三化两拆两改”力度，完成湘潭大道、车站路等道路临街立面整治，确保违法建设零增长。切实治理城市拥堵，建设标准化人车分离工程。继续重拳治理车辆超限超载和交通违法行为。推动燃气管理、户外广告管理等地方性立法。协同推进精美校园建设。加快形成社区和志愿者组织参与的多元化城管机制。力创全国文明城市。全力推进美丽乡村建设。继续打造一批美丽乡村示范片、示范镇和示范村。重点推进城铁、高铁、韶山高速、Ｇ１０７、Ｇ３２０、Ｓ３３０沿线整治，启动韶山灌区水利生态景观带建设，实现美丽乡村由点向面扩展。积极推行“河长制”，加强流域综合治理，打造生态景观共同体。做实全域旅游。完成韶山核心景区提质改造年度任务，启动“最忆韶山冲”文旅综合体项目，推进山市晴岚文旅小镇、昭山水上乐园、湘乡云门广场和水府房车露营基地建设，分步落实全域旅游交通专项规划。支持盘龙大观园创建国家５Ａ级景区，启动彭德怀纪念园、“千里湘江第一湾”景区创建工作。加快湘锰国家矿山公园建设，启动仙女山国家农业公园申报工作。打造齐白石世界级文化品牌。恢复修缮谭政故居、黎氏八骏故居、秋瑾故居等６处名人名居。支持湘潭保税商品中心创建湖南省旅游购物示范点。办好第１４届中国杜鹃花展。

（三）抓实重大项目，发展壮大实体经济。全力推进项目建设。全年组织实施重点项目５００个左右，确保完成投资１０００亿元以上。促进吉利ＳＵＶ生产平台、桑顿新能源２．５期、湘潭电化整体搬迁二期等投产达效，加快舍弗勒湘潭基地、长株潭Ｆ２赛道、湘潭动力配件产业园等建设，推动湖南健康产业园等项目开工。对接国省投资重大工程包和专项建设基金重点支持领域，储备和申报一批重大项目，市本级完成５０个重大项目前期并实现开工。着力重大项目招商。引进一批世界５００强、大型央企、知名民企来潭设立生产基地、研发中心和区域总部。充分发挥行业商会和异地商会作用，紧盯珠三角、长三角、京津冀等重点区域，举办专题招商会。加大市级统筹力度，推动集中招商，年度引进重大项目９０个以上，总投资达４５０亿元以上。推进“项目＋产业”运作模式，依托项目引进新兴产业。实行高位推动和保姆式服务，提升招商项目落地履约率。切实强化要素保障。争取中央预算内资金和专项建设基金支持，加强企业债券申报，力争申报专项３０亿元以上。推进产业发展引导基金、城市发展基金、莲城建设投资基金等有效运作。切实加强金融支持实体经济力度，确保实体经济融资增速达１０％以上。落实国省降低实体经济税费负担、融资成本、用能成本和物流成本相关政策。加大市产业投资基金对实体经济的支持力度。申报省担保基金试点城市，增强市担保基金、风险缓释基金对优质项目和实体经济的融资担保能力。加强用地保障，盘活存量建设用地。加强对投资强度、税收产出等指标的考核评估，促进土地资源节约、合理、高效利用。坚持双向开放、抓牢本土，全力支持本土企业扩大投资、发展壮大。努力实现本土企业枝繁叶茂，外来企业落地深耕，让一切创新创业的活力和发展兴业的资本，在湘潭这片红色的热土上，都享有平等出彩和梦想成真的机会。

（四）着力城乡统筹，推进全域一体发展。加快城乡公共设施一体化建设。全面启动“精美湘潭·畅安交通”三年行动，建成益娄高速湘潭段，尽快拉通湘潭至湘乡、湘潭至花石、韶山至乌石等域内快速干道。加快建设水府大桥。推进干线公路与农村公路有效对接，加强农村道路的建设和管养。完成Ｇ１０７、Ｓ２０８等国省干线公路“白改黑”提质改造。推进公交、供水、燃气城乡一体化。完成冬春水利工程３万处。解决６０００户村民“低电压”问题。提升农业现代化水平。加快国家现代农业示范区建设，切实推进农业供给侧结构性改革。稳定粮食生产能力，发展优质稻１８０万亩。加快培育槟榔、湘莲、茶叶、油茶、花卉苗木等特色产业，加大沙子岭猪品牌开发力度。推进梅林桥、东郊、银田等现代农业示范园建设，支持金薮等万亩油茶示范片和茶恩寺等万亩楠竹示范片发展。设立优质特色农产品展销中心，开展“湘潭十大农产品品牌”评选推介活动。大力发展农产品精深加工，重点支持三旺实业、龙牌酱业等一批农业企业做大做强，提升特色优质农产品供给能力。积极发展农村电商、休闲农业、乡村旅游等产业新业态，培育农业发展新动能。切实推进土地“三权分置”改革，发展适度规模经营，新培育农民专业合作社１００家、发展家庭农场１００家。广辟农业增收致富门路，防止农民增收势头出现逆转。加快县域经济发展。支持天易示范区重点发展食品医药和装备配套产业，湘乡经开区重点发展电子电器和环保电镀产业，韶山高新区重点发展文化创意产业。促进湘潭高新区、经开区等带动县域园区协同发展。做大做强“一县两行”，提升县域金融服务能力。发展县域特色产业和镇村经济，鼓励“一园（区）带多镇”模式，支持有条件的乡镇建设特色工业小区，推进青山桥、金石等工业园建设。加强对乡镇产业发展激励性考核和“扩权强镇”，打造财政收入过千万、过五千万、上亿的经济强镇梯队，形成竞相发展、你追我赶的喜人态势。

（五）坚持问题导向，推进全面深化改革。着力重点领域改革。加快发展资本市场，积极拓展各类直接融资，坚决打击非法集资，防范金融风险。推进国有企业完善现代企业制度，基本完成市属国企战略重组，确保１２家市管企业全部组建到位；完成湘钢、湘电、江南、江麓等驻潭央企省企“三供一业”分离移交年度任务。引导非公有制企业完善法人治理结构，鼓励整体上市、定向增发。推进信用体系建设，加快实现信用信息共享。推进产权保护系列制度改革，进一步激发市场主体活力。加快关键环节改革。进一步精简行政审批事项，推进关联审批、相近类别审批事项“全链条”取消或下放，努力推行“多照合一”“多评合一”“多审合一”等新模式。将“一照一码”登记范围推广到各类市场主体和各个登记环节。严格按照规划设计、工程质量、投资审计同步跟踪要求，切实加强对政府投资项目的全过程监管。全面实行市本级财政全口径预算，实现国库集中支付向乡镇街道全覆盖。建立债务风险管控和应急处置机制，实行政府债务限额管理，加大政府性债务置换力度。编制融资债务预算，加大对平台公司借款和政府支出责任的管控力度，加强政府债券、转移支付、预算收入、政府性基金、国资收益的统筹使用。突出民生事业改革。推进公立医院综合改革，支持医疗健康集团发展，启动市中心医院急诊综合大楼及周边棚户区改造项目，加快市公共卫生服务中心、市中医院整体迁建等项目建设。完善城乡一体的公共服务规划和投入机制，推进城乡社保体系接轨，将进城落户农民纳入城镇住房和社会保障体系。深入推进长株潭“两型社会”试验区第三阶段改革任务。优化城区街道、社区设置。深入推进“三社联动”，将各类矛盾化解在基层、将困难群众的问题解决在基层，将多元化服务供给留在基层，形成治理人人参与、成果人人共享的生动局面。

（六）加大民生投入，共建共享幸福生活。办好民生实事。新增城镇就业５万人，新增农村劳动力转移就业１万人，开展农民素质培训１万人次，重点扶持创业孵化基地和返乡农民工创业基地２０个；新建９个立体停车场，建设人行立体过街设施２０处，设置硬质隔离设施５万余米；改造老旧小区８１个并引导新建小区和改造小区抓好物业管理，提质改造社区公园５个，消除城市照明暗区５００处；扶助全市６０周岁以上失独老人，免费产前筛查孕妇１．７万人，贫困残疾人家庭无障碍改造３００户；为城乡低保、特困供养人员购买意外伤害险，资助农村特困户建房２００户；改造市城区标准化农贸生鲜超市２０个，改扩建蔬菜基地２０００亩；改造、完善村级活动场所和社区公共服务场所各４０处；建设农村公办幼儿园７所；拓宽改造农村公路２５０公里，建设农村公路安保设施２５０公里，改造农村危桥２１座；棚户区（城中村）改造１万套，提质市本级保障性住房小区２０个。既量力而行、又积极有为地办成一批更有温度、更有情怀、更有惠及面、更有获得感的响当当的民生实事。强化社会保障。推进社会保障“一卡通”，加快整合城乡基本养老保险和基本医疗保险。发展社区和居家养老，完成市六医院医养结合项目、乡镇敬老院项目建设。积极推进殡葬惠民改革。强化住房公积金归集扩面，落实省内、跨省缴存异地互认和转移接续。加强农村社区服务体系和综合服务平台建设，建立“三留守”人员关爱服务机制。确保省定贫困村全部摘帽、建档立卡贫困人口全部脱贫，率先探索稳定脱贫、高标准脱贫长效机制，“救急难”等社会救助体系健全高效。发展社会事业。重点抓好高校毕业生、返乡农民工和“去产能”中的分流职工等重点人群的就业，推进库区移民转移就业，城镇零就业家庭保持动态清零。完善企业职工工资正常增长机制。支持驻潭高校争创“双一流”大学，引导校企合作建设“双创基地”和“创新研究院”。积极扶持职业教育，为产业转型升级提供人才支撑。实施教育强市三年行动计划，新增城区义务教育学位２５５０个，新建滴水湖学校，扩建湘钢一中教育集团十二中校区。规范和发展民办教育。建设全国中小学研学旅行实验区。创建全国未成年人思想道德建设先进市。办好湖南省第二届中学生运动会。着力引进和培育一批名医生、名教师和行业名家。加强城乡文化阵地建设，开展“欢乐潇湘·幸福湘潭”等群众文化活动。努力建设一流的城市广播电视台。推行食品药品监管责任网格化、检查格式化、管理痕迹化，保障“舌尖上的安全”。加强环境执法，深入推进大气、水、土壤污染防治。落实湘江保护与治理“一号重点工程”的年度目标任务。严格落实安全生产责任制，坚决杜绝重特大事故发生，切实保障人民群众生命财产安全。深化应急联动机制，坚持打防管控结合，提升社会治安管控效能。推进依法治访、网上信访，及时化解矛盾纠纷。指导做好村居换届工作。落实“七五”普法规划。推进国防建设和双拥工作。认真做好消防、外事侨务、档案、新闻出版、工青妇、供销、移民、气象、水文、地震等各项工作。

（七）科学依法施政，打造人民满意政府。全面树牢“四个意识”，特别是核心意识和看齐意识。落实政府党组“全面从严治党”主体责任，切实加强政府自身建设。建设廉洁政府。始终坚持挺纪在前，切实把“一准则一条例”镌刻在心，融入血脉。巩固“四风”整治成果，扎实推进政府作风建设常态化。带头树立“过紧日子”的思想，严控“三公”经费和一般性支出，切实推进节俭施政、简朴从政，把有限的财力更多地投入到搞建设、促发展、保民生上去。严格压实党风廉政建设“两个责任”。加大审计监督与行政监察力度，严肃查处违法违纪行为，切实做到干部清正、政府清廉、政治清明。建设法治政府。进一步清理行政审批事项，严控新设行政许可。切实推行政府顾问和公职律师制度。落实重大决策合法性审查和“三重一大”决策机制。自觉接受人大的法律监督、工作监督和政协的民主监督，落实重大事项向市人大及其常委会报告和向市政协通报制度，坚决执行市人大及其常委会的决议决定，做好建议提案办理工作，认真听取各民主党派、工商联、无党派人士和人民团体的意见，广泛接受社会监督。建设服务政府。全面提升政府公共服务水平和社会管理能力，加快推进“互联网＋政务服务”，实现部门间数据共享，让群众和企业少跑路、好办事、不添堵，打造更加亲民为民的服务政府。拿出“亲”的正面清单，“清”的负面清单，着力解决“不患亲，患不清”的问题，形成“亲而有别，清而可近”的政商关系。甩开膀子而不是喊破嗓子，为企业解难题、破瓶颈、搭桥梁。更加务实地解决一批招商引资和建设领域的历史遗留问题。建设实干政府。营造干事创业的浓厚氛围，为敢于担当者担当，为敢于负责者负责，为真抓实干者鼓劲，为改革创新者撑腰。

努力提升政府工作人员专业素养和履职能力，锻造一支“机遇面前善谋事、难题面前勇任事、矛盾面前敢扛事、压力面前能成事”的公务员队伍。多搞雪中送炭，少搞锦上添花；多搞轻车简从，不搞前呼后拥；多搞群众有感，不搞数字亮眼；多干打基础、利长远的事，不搞华而不实、哗众取宠的形象工程、政绩工程。建设民本政府。关心基层工作，关爱基层干部，推进政策向基层倾斜、资源向基层集中、成果在基层显现。牢固树立以人民为中心的发展思想，把事关群众切身利益的小事当成大事办、缓事当成急事办、难事当成要事办。把千头万绪的民忧、千差万别的民情、千言万语的民意系在心上，抓在手上，落实在行动上，用实实在在的施政成效，向全市人民交上一份满意的答卷。

各位代表，政贵有恒，惟民惟实。实现市第十二次党代会绘就的宏伟蓝图，关键在于实干。让我们紧密团结在以习近平同志为核心的党中央周围，在省委省政府和湘潭市委的坚强领导下，不忘初心，继续前行，为建设“伟人故里、大美湘潭”，率先迈向基本现代化而努力奋斗！
